# Cultivating Environmentally Sustainable Emergency Departments with *GreenED*

**DOI:** 10.5811/westjem.61385

**Published:** 2026-07-16

**Authors:** Jennifer L. Wood, Alexander Robertson, Tim Spruell, Zoe Steley

**Affiliations:** *GreenED, Royal College of Emergency Medicine, London, United Kingdom; † National Health Service Lothian, Environmental Sustainability Team, Edinburgh, United Kingdom; ‡ National Health Service Fife, Victoria Hospital, Emergency Department, Kirkcaldy, United Kingdom; § University Hospital Sussex, Emergency Department, Brighton, United Kingdom; || Sydney Hospital and Syndey Eye Hospital, New South Wales, Australia

## Abstract

**Introduction:**

Collective leadership within healthcare is critical if we are to deliver on net zero targets and provide high-value, low-carbon care to our patients. However, driving change in systems under considerable strain from crowding, limited resources, and increasingly complex caseloads can be a daunting task and often feels out of reach. In July 2023, the Royal College of Emergency Medicine (RCEM) launched a sustainability initiative to support environmentally sustainable healthcare within emergency departments (ED). This 12-month support and accreditation scheme centers around a framework of 36 actions and has been designed to make sustainability interventions accessible to staff, enabling individuals, groups, and networks to become leaders within their own spheres of influence and elicit measurable changes. The objective of this study was to evaluate the impact of the accreditation scheme over a 2-year period.

**Methods:**

The primary outcome measures were the financial and carbon savings made by participating sites. Secondary outcome measures were derived from a thematic analysis of written submissions, undertaken to identify co-benefits and generate qualitative insights into both barriers to, and enablers of, environmental sustainability interventions within EDs.

**Results:**

Five sites were successfully accredited for Bronze and Silver awards in 2024, and 10 sites for Bronze, Silver and Gold awards in 2025, with combined projected annual savings of £351,366 and 157,724 kg of carbon dioxide equivalent.

**Conclusion:**

To our knowledge, this is the first time that a collaborative, structured, dynamic, and scalable approach to sustainable healthcare in emergency medicine has been used worldwide.

## INTRODUCTION

Sociopolitical factors are now the rate-limiting step in rolling out high-value, low-carbon healthcare.^1^ Collective leadership is, therefore, imperative if we are to stimulate the structural reorganisation and new behaviours within our healthcare system and society that are required to address the increasing presentations of climate crisis patients at the ‘front door’ in emergency medicine (EM).^2^,^3^ As a threat multiplier, the climate crisis will continue to exacerbate existing health and social inequalities for future generations and challenge our already struggling systems.

In response to the existential threat of climate change, the UK’s National Health Service (NHS) committed to reaching net zero emissions in October 2020, becoming the world’s first health service to do so.^4^ However, the healthcare system itself compounds this crisis, generating around 4–6% of the UK’s total carbon footprint.^5^, ^6^ Some examples include the overuse of non-sterile gloves, which are derived from fossil fuels and often flown or shipped from Asia; meter dose inhalers, which contain a hydrofluoroalkane gas 1,300 times stronger than carbon dioxide and whose plastic, derived from fossil fuels, is currently difficult to recycle; and low-value radiology and pathology tests, which all have financial and environmental concerns, as well as patient safety concerns when inappropriately utilized.^7^–^10^

We commend NHS Scotland’s Climate Change Risk Assessments and Adaptation summary report and NHS England’s Green Plan guidance which help to identify the next steps in decarbonising the NHS. However, amid crowding, staff shortages, and increasingly complex presentations, many healthcare workers lack the time, knowledge, support, and confidence in their ability to enact behavioural and systems-level change.^11^–^13^

The Royal College of Emergency Medicine (RCEM) launched a sustainability initiative called *GreenED* in July 2023, after a pilot study in England from 2021–2022.^14^ This framework of actions has been designed by emergency clinicians to help emergency department (ED) staff in high resource settings to reduce environmental impacts of clinical practice and departmental operations whilst quantifying carbon and financial savings associated with transitioning to high-value, sustainable models of patient care. To our knowledge, this is the first time that a collaborative, structured, dynamic, and scalable approach to sustainable healthcare in EM has been used. This brief report will discuss the scheme which consists of the development of the framework and share insights from the EDs that were accredited at Bronze, Silver, and Gold levels in 2024 and 2025.

### What is it?

**Figure 1:**
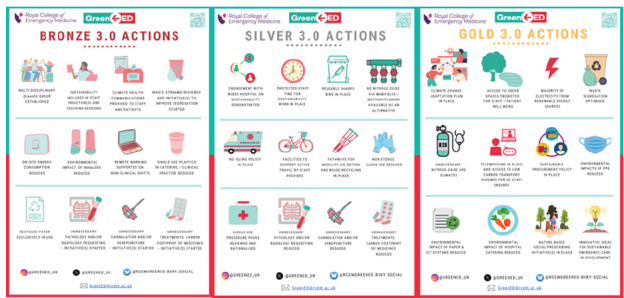
*GreenED* Framework 3.0 Bronze, Silver and Gold levels (updated May 2025). This figure depicts the actions in Framework 3.0, separated into Bronze, Silver and Gold, that emergency departments (ED) can undertake to make their ED more environmentally sustainable over the short-term, mid-term, and long-term. respectively.

*GreenED* is a 12-month support and accreditation scheme that consists of evidence-based actions, structured into three levels by order of feasibility —immediate changes (Bronze); mid-term changes (Silver); and long-term changes (Gold)—to make an ED more environmentally sustainable (Figure 1). Sites typically progress through these levels in a stepwise manner.

Whilst some resources are open access, sites can pay a fee (£2,200 as of 2026) to access further resources. These include an online portal with customized collated resources which equips participants to design and implement practical departmental changes, and monthly troubleshooting meetings facilitated by clinicians with sustainable healthcare expertise, which help to share achievements and identify barriers to action implementation. This iterative process enables participants to become leaders in their own spheres of influence, feeding innovative case studies back into the scheme to empower others and grow the evidence base for environmentally sustainable emergency healthcare.

### How was it created?

In 2019 the RCEM established the Environmental Specialist Interest Group (ESIG) and tasked the appointed volunteers with developing ways to make daily clinical practice more environmentally sustainable. A working group of emergency physicians and advanced care practitioners from hospitals in the UK was assembled to discuss environmental sustainability actions which could be enacted in an ED setting.

With potential actions identified, ESIG and the RCEM Quality Team collaborated with the founders of the Laboratory Efficiency Assessment Framework (LEAF), a certification in sustainable laboratory operations managed by University College London, on the proposed initial *GreenED* framework, termed Framework 1.0.^15^ An induction webinar was held in November 2021 for emergency clinicians interested in trialing the framework as pilot sites where the background to the Framework 1.0 was shared along with its potential to contribute towards the target of NHS net zero 2040 targets. Ten sites in England registered for an eight-month pilot. Initial seed funding of £83,000 from a healthcare public body allowed for the development of the framework and pilot project to be undertaken. Sites were provided with digital monthly catch ups with members of the *GreenED* team, the Framework 1.0, infographics, and a project folder where they could submit required reports (following a standardised reporting format) and evidence of their actions in the form of reports, photos, videos, slide decks and posters.

Accreditations standards and minimum evidence requirements were developed with ESIG, LEAF and the RCEM quality directorate. Submissions were examined by members of the ESIG and LEAF teams at the end of the eight-month period and included discussions with the site leads. Eight of the sites were accredited with Bronze level awards. Feedback from sites and teams involved in the pilot was used to improve and develop the *GreenED* Framework 2.0 which was reviewed and endorsed by a healthcare public body. Improvements included the building of a customized online portal hosted by the RCEM and customized carbon calculators, along with some minor adjustments to the framework (difficult-to-attain actions were moved to Silver levels), and it was launched by the RCEM in July 2023 for sites in the UK to voluntarily sign up to.

## METHODS

Participating groups worked through the *GreenED* Framework 2.0 in ‘communities of practice’ to enable collaboration and troubleshooting. Using the portal, sites submitted qualitative data about achieving actions and describing barriers faced, along with quantitative data using the customized portal carbon calculators for certain actions, including adjusting computer monitor brightness, low-carbon staff travel, using recycled paper, energy efficiency of light emitting diode (LED) lights, and use of cannulas. Carbon savings are measured using carbon dioxide equivalent (CO_2_e), a universally recognised metric that expresses the warming impact of all greenhouse gases in terms of the equivalent amount of CO_2_. Financial savings for each action were reported by sites according to the local procurement data in their ED in pound sterling. Savings categories were defined as Patient Pathways, Procurement, Energy, Waste, and Travel.

Patient pathways included savings made in cannulation pathways, pathology/radiology savings, reduced non-sterile glove use, and switching from nitrous oxide to methoxyfluorane in the ED. Methoxyfluorane (Penthrox) has a longer duration of analgesia compared to nitrous oxide, can be delivered via a small hand-held inhaler, and has a significantly lower CO_2_e than nitrous oxide. Other pathways include the following: procurement savings related to recycled paper and reusing mobility aids; waste savings related to correct waste streaming between domestic, clinical, and recycling bins; energy savings from reducing monitor brightness/reducing time to idle, switching to LED lights and using sensors to reduce use in low-use areas; and travel savings related to staff changing modes of transport to work or by working remotely on non-clinical days.

Assessors reviewed the quantitative savings submitted by sites along with the evidence submitted for these savings. Quantitative evidence was then sorted into savings categories as defined above and extrapolated to calculate projected annual financial and carbon savings. If sites were unable to produce sufficient evidence for their calculations (such as incomplete procurement data or savings which were part of a Trust-wide initiative as opposed to an ED-only initiative) these savings were not included in the final calculations in Tables 1 and 2.

Institutional governance approval for the thematic analysis was obtained by the Clinical Effectiveness Team in a UK district general hospital. Using peer-reviewed standards, a pool of clinicians who assessed the submissions, performed a thematic analysis on the evidence from the five sites that submitted for accreditation in 2024. Each submission was reviewed separately by two assessors who created codes and identified initial themes. Themes from the written submissions were then shared with and collated by the GreenED team. The team also compiled the available data on carbon and financial savings from all sites that submitted in 2024 and 2025.

## RESULTS

Summative quantitative results are listed in Table 1, highlighting projected financial savings per year that can be made whilst maintaining high-value, low-carbon clinical care, whilst Table 2 shows the individual sites projected carbon and cost savings made through the specific actions they chose to target.

The GreenED actions resulted in clear financial and carbon savings for EDs. Quantitative savings are a conservative projection due to limitations on data-gathering abilities in busy departments. This is being examined and improved for Framework 3.0. Not all departments were able to undertake comprehensive audits or submit projected savings due to time and capacity constraints, and results are likely an underestimate.[Fig f2-wjem-27-1105]

The thematic analysis revealed 28 different themes which are showcased in Table 2. We discuss particular theme highlights below.

### Theme Highlights

#### Sustainability leads can effect change beyond the emergency department

Sites reported that education and leadership from the GreenED team contributed to departmental level changes and had a positive impact on department culture, leading to sustainable changes in work and at home. Sites found that GreenED actions complemented Trust sustainability plans, and sustainable working practices were ‘shared and taken up by other areas of the hospital’. Extensive co-operation between hospital specialties and departments was reported. One site acknowledged that ‘conversations on sustainability that begin in [the] ED can be continued in primary care’, highlighting the interface between ED and primary care. Another site reported that after ‘the Sustainability fellow...for ED was employed...the momentum for sustainability work took off’, highlighting that strong environmental leadership within a collective can influence entire organisations.

#### Multidisciplinary and inclusive teams are the most effective

‘We have a large Initiative working group which meets up every three months. Our group includes doctors, nurses, advanced clinical practitioners, emergency nurse practitioners, managers, pharmacists, IT managers and sustainability managers.’

All sites reported that the success of the GreenED teams relied on inclusive and multidisciplinary teamwork; colleagues from different departments gravitated towards the group, breaking down professional silos. Many teams are now in regular contact with professionals in estates, domestics, sustainability as well as other community and hospital specialties.

#### The Initiative saves money and carbon

‘We changed our screen brightness settings from 80% to 50%, installed a night mode to our screens and have communicated with our IT teams about changing our screensaver times. This is projected to save 3,500 kg CO_2_e per year.’

All actions were designed to meet the triple bottom line, used to describe the intersection of improved patient care, improved environmental sustainability, and financial savings. Addressing systemic waste by optimising IT settings, enhanced waste segregation as well as reducing low-value care (such as unnecessary cannulation) resulted in reductions in carbon emissions, whilst also resulting in financial savings. If scaled across the hospital sites, the interventions could lead to significant cost and carbon savings.

#### Systemic barriers to progress were identified across a variety of actions

‘We have struggled to meet this target. Salbutamol DPI [dry powder inhaler] is not available on our regional formulary’.’

Systemic barriers to specific sustainability actions were identified by sites during the monthly catch. ups and accreditation process. Commonly encountered barriers included not having a kitchen to wash reusable cups, as well as dry powder inhalers not being available on regional formularies. Departments would struggle to overcome these barriers alone, but by identifying them through the accreditation process, the RCEM’s ESIG was able to advocate for changes at regional and national levels.[Table t3-wjem-27-1105]

## DISCUSSION

Leadership, in the case of sustainable healthcare, is making change accessible, thus enabling individuals, groups and networks to become leaders in their own right. This produces collective leadership which has the capacity to activate socioeconomic tipping points and build alignment and commitment into organizations.^3^, ^16^ Analysis of the 15 sites achieving accreditation revealed that the RCEM *GreenED* initiative helped to facilitate staff action by providing structured guidance, resulting in coordination and continued effort in sustainable healthcare actions. The most successful sites had teams consisting of multidisciplinary members, and their improvements led to changes in the wider hospital, not just in their department. Through the site submissions, we have been able to identify systemic barriers, and this evidence has allowed us to advocate for change at national and international levels.

Though an underestimate due to limitations of data collection, between 2024–2025 the 15 sites saved a total of £351,366 and 157.72 tonnes of CO_2_e per year, demonstrating that environmentally sustainable healthcare is beneficial for patients, staff, finances, and the planet. This is an underestimate, as some data were excluded due to insufficient evidence from sites due to participant capacity constraints, difficulties sourcing data due to limitations of the systems that we operate in or, in one case, because the savings made were felt to be attributable to a hospital-wide scheme of mobility aid reuse, as opposed to the ED spurring this itself. However, whilst a modest reduction in both pounds sterling and CO_2_e, it is the first quantified reduction using a tailored sustainability initiative in an ED setting that we know of. Participants were able to achieve this with no protected or paid time to carry out these initiatives, and whilst prioritizing emergency care in the context of departments which are facing record levels of ambulance waits and crowding leading to patient harm, moral injury, and staff turnover.^17^,^18^,^19^ As some of the first to carry out this work, we also wish to highlight that some systems and items have not had life cycle analyses carried out, which further complicates the ability to calculate carbon and financial savings. With participants feedback, data collection for CO_2_e and finances is developing rapidly and improving for Framework 3.0 (Figure 1), which was recently published on our website.^14^

Through the thematic analysis, we discovered that *GreenED* enables staff to adopt leadership roles within their circles of influence and can result in collective leadership at an organisational level. Sites reported the positive deconstruction of professional silos, a key factor in effecting behavioral change and scaling sustainable healthcare.^20^

## LIMITATIONS

There were several limitations to our evaluation. The first limitation is that participation in GreenED is voluntary, thus making the study vulnerable to self-selection bias. Sites that have chosen to participate are likely to have a higher level of environmental awareness and motivation than non-participating sites. Consequently, findings may overestimate the ease with which findings could be replicated more broadly. Similarly, the joining fee will limit some sites from being able to participate in the scheme. Whilst most individuals overseeing the management of GreenED are voluntary, resources are required for the RCEM to run the portal and website and cover RCEM quality team time. To improve this, we have approached some governments to ask if they would consider funding the joining fee for sites in a particular country (e.g., Wales) to remove that barrier.

The second is the size of the evaluation; 15 sites took part in the accreditation scheme between 2024–2025 from England and Wales, which is relatively small. However, these are sites of different sizes with different demographics, and each site was able to follow the GreenED framework and execute the actions to a satisfactory level for accreditation. We have 49 sites that have signed up for Framework 3.0 in 2026 which will allow us to analyse a far greater collection of data and a more accurate estimation of CO_2_e and financial savings.

Lastly, data collection methods could have been improved. Patient safety and departmental requirements will always come first, and that can make consistent data collection difficult during a busy ED shift; our financial and CO_2_ predicted savings are all underestimates because of this. Improved data collection systems are being developed for Framework 3.0.

## CONCLUSION

A systematic and supported approach to environmentally sustainable emergency medicine can elicit rapid decarbonisation and lead to substantial annual financial savings. Applying the guiding principle of ‘Think big, start small, scale quickly’, GreenED has rapidly expanded to provide leadership at an international level. An Australian pilot (13 sites) is in its final stages, and the 2025/26 programme has expanded to Ireland (4 sites), with 32 sites from England and Wales taking part and interest being expressed from EDs in mainland Europe. We are also investigating the feasibility of a modular approach to the frameworks, allowing sites to substitute actions from one level into another to give greater flexibility.

We are currently developing GreenED International which we envision being used as an internationally recognised benchmark for environmentally sustainable emergency care in high resource settings. Rapid decarbonisation of healthcare requires that best practices are shared, and we hope that other countries and specialties can use the initiative as a blueprint to adapt and grow environmentally sustainable and resilient healthcare beyond the ED.

## Figures and Tables

**Figure 2 f2-wjem-27-1105:**
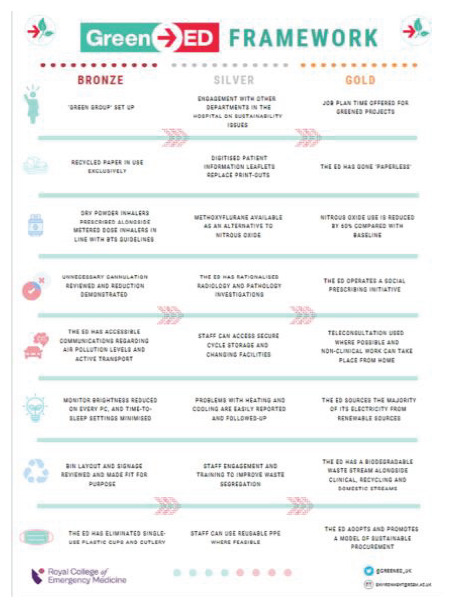
An original *GreenED* Infographic which has since been developed into GreenED Framework 3.0 (see Figure 1). *ED*, emergency department. *ED*, emergency department.

**Table 1:** Projected quantitative savings rounded to the nearest pound (£) and kilogram from emergency departments using *GreenED* Framework 2.0.

	Patient Pathways (£)	Procurement (£)	Waste (£)	Energy (£)	Total	Total CO_2_ (kg)
2024 Projected Savings per year	£45,196	£7,857	£9,000	£1,505	£63,559	37,962 kg
2025 Projected Savings per year	£235,350	£7,010	£11, 244	£34,203	£287,807	119,762 kg
Total Savings	£280 546	£14, 867	£20, 244	£35, 708	£351, 366	157,724 kg

*CO_2_*, carbon dioxide.

**Table 2:** Site-specific projected annual savings in kilograms and pounds sterling (£) from emergency departments using *GreenED* Framework 2.0.

	Site 1 CO_2_e (kg)	Site 1 Cost (£)	Site 2 CO_2_e (kg)	Site 2 Cost (£)	Site 3 CO_2_e (kg)	Site 3 Cost (£)	Site 4 CO_2_e (kg)	Site 4 Cost (£)
Procurement	0	0	0	0	0	0	0	0
Waste	0	0	0	0	0	0	N	9000
Energy	433.13	497	0	0	3500	N	0	0
Pathways	16300	20,000	4152.51	7215.2	2974	53.28	1474.2	18000
Travel	0	0	0	0	0	0	0	0
Total	16733.13 kg	£20,497	4152.51 kg	£7215.2	6474 kg	£53.28	1474.2 kg	£27000
	**Site 5 CO** **_2_** **e (kg)**	**Site 5 Cost (£)**	**Site 6 CO** **_2_** **e (kg)**	**Site 6 Cost (£)**	**Site 7 CO** **_2_** **e (kg)**	**Site 7 Cost (£)**	**Site 8 CO** **_2_** **e (kg)**	**Site 8 Cost (£)**
Procurement	4700	7785	1184	1235	0	0	0	0
Waste	0	0	0	0	0	0	1916.25	728
Energy	959.91	1008.37	745	1620	516	469.47	157.18	14723
Pathways	3468	N	6967	14852	1076	N	273	19466
Travel	0	0	9397	N	0	0	1910	N
Total	9127.91 kg	£8793.37	18293 kg	£17707	1592 kg	£469.47	4256.43 Kg	£34917
	**Site 9 CO** **_2e_** ** (kg)**	**Site 9 Cost (£)**	**Site 10 CO** **_2_** **e (kg)**	**Site 10 Cost (£)**	**Site 11 CO** **_2_** **e (kg)**	**Site 11 Cost (£)**	**Site 12 CO** **_2_** **e (kg)**	**Site 12 Cost (£)**
Procurement	619	1943	720	N	0	0	0	0
Waste	0	0	109	3276	18488	7240	0	0
Energy	0	0	48	78	14800	16645	495	450
Pathways	0	0	N	65700	1281	2200	0	0
Travel	0	0	0	0	0	0	0	0
Total	619kg	£1943	877	£69054	34569	£26085	495	£450
	**Site 13 CO** **_2_** **e (kg)**	**Site 13 Cost (£)**	**Site 14 CO** **_2_** **e (kg)**	**Site 14 Cost (£)**	**Site 15 CO** **_2_** **e (kg)**	**Site 15 Cost (£)**		
Procurement	117	832	0	0	0	0		
Waste	0	0	0	0	0	0		
Energy	315	217.29	0	0	0	0		
Pathways	0	0	27894	82932	23091	53200		
Travel	0	0	0	0	0	0		
Total	432	£1049.29	27894	£82932	23091	£53200		

The letter ‘N’ signifies that savings were submitted by sites but did not meet evidence standards and were excluded from the final dataset.

*CO_2_e*, carbon dioxide equivalent.

**Table 3 t3-wjem-27-1105:** Qualitative themes identified from thematic analysis of the Initiative site submissions 2024–2025.

Thematic analysis from submissions	
Sustainability work was already underway in some EDs before starting GreenED accreditation.	Staff could still participate in the Initiative despite the limited time available.
The accreditation has led to innovative sustainability work both within and out of the ED.	The initiative saves money and carbon emissions.
Implementing clear signage on bins and teaching sessions on waste segregation are useful ways to instigate change.	The initiative has reduced single-use plastic waste.
Digital communication including Microsoft Teams, email, social media, and video are invaluable tools for a successful project.	Showing strong environmental leadership by having sustainability specific roles leads to quicker change both in the ED and wider hospital and can even change hospital culture.
Posters, notice boards and suggestion boxes are well used to exchange sustainability information with patients and staff.	ED staff already have concerns about our impact on the environment; listening to these concerns helps inform sustainability action and increases engagement.
GreenED teams are most effective when multidisciplinary, inclusive and with good working relationships with teams in the wider hospital.	Introducing new waste streams and rearranging bin placement were needed for departments to improve their waste segregation.
GreenED teams see that a close working relationship with Trust sustainability managers helps to progress projects.	Small-scale changes can empower staff and lead to positivity within the workforce.
Departments have used the champion role and badges to identify core GreenED members.	Changing firmly held ideas and behaviors about sustainability can be difficult.
Prioritising the Initiative by adding it as a standing agenda item both departmentally and at Trust level saw improved project success.	Initiative calculators help to quantify carbon and cost savings.
Regular meetings of the Initiative group are important to keep momentum going and check on progress.	Going through the GreenED process has spurred improvements in the sustainability of the built environment.
Teams have identified systemic barriers to progress in a variety of actions.	Remote working is well embedded in EDs and reduces the carbon footprint of travel to the hospital.
The initiative aligns with Trust sustainability plans and acts as a trigger for innovative NHS wide sustainability changes.	Listening to staff feedback on sustainability increases engagement.
Further work needs to be done to determine patient perspectives on sustainability.	Reductions in unnecessary cannulation saves time and improves patient experience.
Quality improvement methodology was used to implement changes.	There are a variety of sustainability resources and initiatives in the wider NHS that can be used to support work on GreenED.

In total, 28 themes were identified in the thematic analysis from the reports generated by the five sites who submitted for accreditation in 2024.

*ED*, emergency department; *NHS*, National Health Service.
